# The Quantum-Volume and Bragg-Williams Equations of State

**DOI:** 10.1021/acs.jpcb.6c02888

**Published:** 2026-07-25

**Authors:** Per-Olof Åstrand

**Affiliations:** Department of Chemistry and Biomedical Science, 8018 NTNU - Norwegian University of Science and Technology, Trondheim NO-7481, Norway

## Abstract

The quantum-volume (qv) and Bragg-Williams equations of state, as derived from a lattice gas model, are exploited. The qv equation of state includes a temperature-dependent excluded volume related to the thermal wavelength included in the translational contribution to the molecular partition function, but apart from this contribution intermolecular interactions are not included. The qv equation of state has the correct limiting behavior both in the limit of an ideal gas and in the packing-density limit. It also includes a nonclassical contribution to the internal energy and thereby a many-particle contribution to the kinetic energy, and it supports the view that it is both the position as well as the momentum of a system of particles that are distributed over the volume to maximize the multiplicity of the system. The qv equation of state is extended with intermolecular interactions through the Bragg-Williams mean-field approach, including also a hard-core contribution. For a one-component lattice gas, analytical expressions are presented for the pressure and the critical point as well as for the internal energy, the isochoric heat capacity, and the chemical potential, and results are presented for some simple systems. Given its relative simplicity, essentially on a textbook level, the model shows promising results, in particular for the temperature dependence of the isochoric heat capacity that shows a maximum close to the critical temperature. The presented model is supposed to serve as a foundation for further model extensions as well as for the development of interpretable analytical models in molecular thermodynamics for more complex systems and processes.

## Graphical abstract

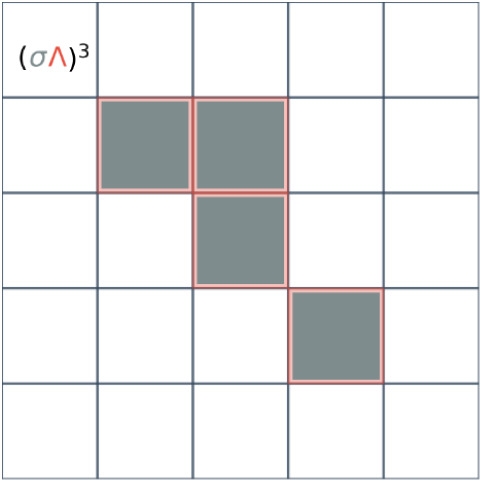

## Introduction

Lattice models have over the years been used extensively in molecular thermodynamics and is a powerful tool for introducing basic concepts in statistical thermodynamics where the chemical potential is the central driving force as solutions, alloys, adsorption to surfaces, and polymer conformations.^1–4^ Recent work adopting lattice models includes diverse areas as high-entropy alloys,^5^ adsorption processes,^6^ supercritical processes,^7^ and Flory–Huggins models for phase separation.^8^ A central aspect of a lattice model is the configurational entropy, *S*_conf_, given by the multiplicity, *W*, through Boltzmann’s entropy law, *S*_conf_ = *k*_B_ln*W*, where *k*_B_ is Boltzmann’s constant. The multiplicity is the number of ways a set of indistinguishable particles (molecules) can be placed on a lattice. The concept of configurational entropy has, however, been questioned and discussed in detail mainly from a teaching perspective.^9–11^ The fundamental reason is mainly that given that we have only one (type of) entropy (which may have several contributions), it is difficult, if not done properly (see e.g., pages 263–264 in ref 12), to at the same time and concisely explain both the configurational entropy, which may be regarded as a positional entropy, and the Clausius entropy, which is related to heat.

In a recent paper, which is the basis for this work, we examined lattice gas models as used in molecular thermodynamics.^13^ The main finding was that the configurational entropy needs to be merged with the molecular translational entropy for a consistent model, and as a consequence, the configurational entropy contribution to the partition function must in the limit of an ideal gas give the translational partition function for *N* particles. The reason is that otherwise, when deriving the pressure, we get the ideal gas law twice, which is once too many. Consequently, the volume of a lattice point for a lattice gas is Λ^3^, where Λ is the regular thermal wavelength for a particle with the mass *m* given by

1 $$\Lambda =\frac{h}{\sqrt{2\pi mk_{B}T}}$$

where *h* is Planck’s constant and *T* is the temperature. Thereby, the volume of a lattice point depends on the temperature. Although Λ^3^ has the role of a physical excluded volume, its origin is that a particle also has a wave-like nature. From a modeling point of view, Λ^3^ arises from an approximate calculation of the translational partitioning function from a quantum-mechanical model system, a particle-in-a-box. One could imagine other models for the translational partition function, but the key point here is that the translational partition function needs to match the configurational partition function to avoid an overcounting in the calculation of the pressure. To use Λ^3^ as the volume of a lattice point is a concept discussed by Yu, where Λ^3^ was termed the *quantum volume*.^14^ To some extent, this is also in analogy with the derivation of the Sackur-Tetrode equation using information theory.^15^

In addition, a derivation of the van der Waals equation of state using a lattice model was revived,^13^ where the interaction term for the lattice gas was obtained using the Bragg-Williams approximation.^16–18^ To adopt Λ^3^ as the volume for a lattice point, and thereby including an additional temperature dependence in the partition function, results in a modified temperature dependence for the internal energy, *U*, beyond the classical term for the kinetic energy, *U* = 3*Nk*_B_ *T*/2. For the isochoric heat capacity, *C*_*V*_, that is independent of temperature, *C*_*V*_ = 3*Nk*_B_/2, both for an ideal gas as well as for the traditional van der Waals equation of state, the quantum-volume approach results in a temperature-dependent *C*_*V*_. Note that this additional temperature dependence is obtained also without including intermolecular interactions apart from the excluded volume of Λ^3^ per particle, and this term may therefore be regarded as a term describing correlation of the motion of the molecules or as a many-body contribution to the kinetic energy. It also implies that the configurational entropy, sometimes regarded only as a positional entropy by distributing the particles over a lattice, actually should be regarded as a distribution of both the position and momentum of the particles over the lattice in line with the work by Ben-Naim.^19^

As an intermediate result in the derivation of the van der Waals equation of state from a lattice gas model, we also obtained an equation of state termed the Bragg-Williams equation of state that has fewer approximations than the van der Waals equation of state,^13^ and therefore the Bragg-Williams equation of state is investigated in more detail in this work. Analytical expressions for the critical point, the internal energy, *U*, the isochoric heat capacity, *C*_*V*_, and the chemical potential, μ, are derived with the temperature dependence provided by the quantum-volume approach. The Bragg-Williams equation of state is here extended by a hard-core potential that in conjunction with the quantum volume, Λ^3^, gives the volume of a lattice point.

## Theory

### Quantum-Volume Equation of State

Here, we take one step back and regard a system of *N* molecules distributed on *M* lattice points without intermolecular interactions, but with an excluded volume of Λ^3^ per molecule. The configurational entropy, *S*_conf_ was derived from the multiplicity, *W*, as^13^

$$\begin{array}{ll} S_{\mathrm{conf}} & =k_{B}\ln W=k_{B}\ln\frac{M!}{N!\left(M-N\right)!}\\ & \approx k_{B}(\ln\frac{1}{N!}+M\ln M-M-\left(M-N\right)\ln\left(M-N\right)+M-N)\\ & =k_{B}(\ln\frac{1}{N!}+\left(M-N\right)\ln M+N\ln M-\left(M-N\right)\ln\left(M-N\right)-N)\\ & =k_{B}\left(\ln\frac{1}{N!}+N\ln M-\left(M-N\right)\ln\left(1-\frac{N}{M}\right)-N\right)\\ & =k_{B}\left(\ln\frac{M^{N}}{N!}+N\left(\left(1-\frac{M}{N}\right)\ln\left(1-\frac{N}{M}\right)-1\right)\right)\\ & =k_{B}\left(\ln\frac{V^{N}}{b^{N}N!}+N\left(\left(1-\frac{V}{Nb}\right)\ln\left(1-\frac{Nb}{V}\right)-1\right)\right) \end{array}$$ 2

where we first used Stirling’s approximation for *M*! and (*M*-*N*)!, but not for *N*!. In the last step, the volume of the system, *V*, is proportional to the number of lattice points, *V* = *bM*, where *b* is the volume of a lattice point, which follows from the merging of the configurational and translational partition functions in the ideal gas limit.^13^ The model discussed in this work is thus restricted to Maxwell–Boltzmann statistics for classical particles. Since we disregard intermolecular interactions, its contribution to the internal energy is zero, and therefore the contribution from a lattice model to the Helmholtz free energy becomes *F* = -*TS*_conf_. Utilizing that *F* = −*k*_B_ *T*ln*Q*, where *Q* is the partition function, we define the quantum-volume (qv) equation of state in terms of the partition function as

3 $$\begin{array}{ll} \ln Q^{\mathrm{qv}} & =\ln\frac{V^{N}}{b^{N}N!}+N\left(\left(1-\frac{V}{Nb}\right)\ln\left(1-\frac{Nb}{V}\right)-1\right)\\ & =\ln\frac{V^{N}}{\Lambda^{3N}N!}+N\left(\left(1-\frac{V}{N\Lambda^{3}}\right)\ln\left(1-\frac{N\Lambda^{3}}{V}\right)-1\right) \end{array}$$

where we in the second step adopted *b* = Λ^3^. Because

4 $$\underset{x\to 0}{\lim}\frac{1}{x}\ln\left(1-x\right)=-1$$

the translational partition function for *N* molecules, and thereby the correct ideal gas limit, is obtained when *N*/*V* approaches zero. An equation of state is often presented as *p*(*V*,*T*), and the pressure, *p* ^qv^, for the qv equation of state is given as

5 $$p^{\mathrm{qv}}=k_{B}T{\left(\frac{\partial \ln Q^{\mathrm{qv}}}{\partial V}\right)}_{T,N}=-\frac{k_{B}T}{\Lambda^{3}}\ln\left(1-\frac{N\Lambda^{3}}{V}\right)$$

The ideal gas law is retrieved by adopting the Maclaurin series, ln­(1-*x*) ≈ -*x*, valid when *N*/*V* approaches zero.

The pressure for the qv equation of state in eq 5 have a divergence at the packing density limit, *V* = *N*Λ^3^, which is an important feature of an equation of state and has led to modifications of other equations of state.^20,21^ Equally important, the partition function in eq 3 does not have this divergence because (1–1/*x*) ln­(1-*x*)→0 when *x* → 1.

In addition, in the derivation of the van der Waals (vdW) equation of state from a lattice model, where the pressure for the qv equation of state in eq 5 is obtained as an intermediate result, the correct packing density limit is lost because of the two subsequent approximate steps applied on eq 5 (see pages 481–482 in ref 4),

6 $$\ln\left(1-x\right)\approx -x-\frac{x^{2}}{2}\approx \frac{-x}{1-\frac{x}{2}}$$

where we in the first step used the Maclaurin series for ln­(1-*x*) ≈ -*x* - *x* ^2^/2 and second the approximation (1 + *x*/2) ≈ 1/(1-*x*/2). With *x* = *N* Λ^3^/*V*, it leads to the excluded volume contribution to the vdW equation of state of as *N k*_B_ *T*/(*V* - *N* Λ^3^/2), but rather with *b* = Λ^3^/2 than *b* = Λ^3^ where only the latter gives the correct packing-density limit. It is evident that the excluded volume contribution to the vdW equation of state has an error of a factor of 2 for the packing density limit as compared to eq 5 and in line with previous results.^20^ The reason is that both approximations in eq 6 are valid for *x* → 0, but not for *x* → 1. This result is a strong argument for keeping and further investigating the BW equation of state over the vdW and similar equations of state.

Since we with *b* = Λ^3^ introduce an additional temperature dependence, it is of interest to investigate some common properties obtained by differentiating the partition function with respect to the temperature as the internal energy, *U*, and the isochoric heat capacity, *C*_*V*_, and compare to the results for an ideal gas. We will in the sections below investigate another choice for a temperature-dependent lattice point volume, *b*, and we will therefore as an intermediate step carry out a general derivation considering *b*(β) with β = 1/*k*_B_ *T*. The internal energy becomes by starting from eq 3

7 $$U^{\mathrm{qv}}=-{\left(\frac{\partial \ln Q^{\mathrm{qv}}}{\partial \beta }\right)}_{V,N}=-\frac{Vb^{\prime }\ln\left(1-\frac{Nb}{V}\right)}{b^{2}}=-\frac{3k_{B}TV\ln\left(1-\frac{N\Lambda^{3}}{V}\right)}{2\Lambda^{3}}$$

where *b*’ = *∂b*/*∂*β = 3Λ^3^/2β and in the second step, *b*’/*b* ^2^ = 3*k*_B_ *T*/2 Λ^3^ when *b* = Λ^3^. It is found that *U* ^qv^ diverges at the packing density limit, *V* = *Nb* or *V* = *N*Λ^3^. Because Λ → 0 at high temperatures, it is expected that the ideal gas result is obtained for *T* → ∞. This is obtained by rewriting eq 7 as

8 $$U^{\mathrm{qv}}=-\frac{3Nk_{B}T}{2}\frac{V}{N\Lambda^{3}}\ln\left(1-\frac{N\Lambda^{3}}{V}\right)\approx \frac{3Nk_{B}T}{2}$$

and again adopting eq 4. Importantly, the result is obtained both for an ideal gas, *N*/*V* → 0 at any temperature and at high temperatures, when Λ^3^ → 0, at any number density, *N*/*V*. Since the ideal gas limit in eq 8 is the average kinetic energy of the system, *U* ^qv^ should be interpreted as a correction to the kinetic energy resulting from an excluded volume, Λ^3^, of each molecule. *U* ^qv^ thus includes a correction term that describes the correlated motion of the molecules that is not included if the “classical” partition function is used as a starting point.

The isochoric heat capacity, *C*_*V*_, is in turn obtained as

9 $$C_{V}=-k_{B}\beta^{2}{\left(\frac{\partial U}{\partial \beta }\right)}_{V,N}=k_{B}\beta^{2}\left(\frac{V\left(b^{\prime \prime }b-2b^{\prime 2}\right)}{b^{2}}\ln\left(1-\frac{Nb}{V}\right)-\frac{Nb^{\prime 2}}{b^{2}\left(1-\frac{Nb}{V}\right)}\right)$$

where *b*” = *∂* ^2^ *b*/*∂ β* ^2^. To obtain *C*_*V*_ for the qv equation of state, *b* = Λ^3^, we need

10 $$\frac{b^{\prime \prime }b-2b^{\prime 2}}{b^{2}}=-\frac{15}{4\Lambda^{3}\beta^{2}}$$

with *b*” = 3 Λ^3^/4 β^2^ and

11 $$\frac{b^{\prime 2}}{b^{2}}=\frac{9}{4\beta^{2}}$$

resulting in

12 $$C_{V}^{\mathrm{qv}}=-\frac{15k_{B}V}{4\Lambda^{3}}\ln\left(1-\frac{N\Lambda^{3}}{V}\right)-\frac{9Nk_{B}}{4\left(1-\frac{N\Lambda^{3}}{V}\right)}$$

We note that both the terms for the isochoric heat capacity diverge at the packing density limit, *V* = *N*Λ^3^. The correct limit, both for *N*/*V* → 0 and *T* → ∞, is, as for the internal energy, *U* ^qv^, obtained by using eq 4,

13 $$C_{V}^{\mathrm{qv}}\approx \frac{15Nk_{B}}{4}-\frac{9Nk_{B}}{4}=\frac{3}{2}Nk_{B}$$

The summation of essentially the same two terms, 15/4–9/4, to reach the ideal gas relation for the heat capacity has also been obtained by using a model based on the Shannon entropy and discrete states.^22^

The chemical potential for the qv equation of state, μ^qv^, is obtained from eq 3 as

14 $$\begin{array}{ll} \mu^{\mathrm{qv}} & =-k_{B}T{\left(\frac{\partial \ln Q^{\mathrm{qv}}}{\partial N}\right)}_{T,V}\approx -k_{B}T\left(\ln\left(1-\frac{N\Lambda^{3}}{V}\right)+\ln\frac{V}{N\Lambda^{3}}\right)\\ & =-k_{B}T\ln\left(\frac{V}{N\Lambda^{3}}-1\right) \end{array}$$

where Stirling’s approximation, ln *N*! ≈ *N* ln *N* - *N*, has been used. In the limit, *N*/*V* → 0, μ^qv^ is reduced to the correct expression for an ideal gas as obtained from the translational contribution to the molecular partition function,

15 $$\mu^{\mathrm{id}}=-k_{B}T\ln\frac{V}{N\Lambda^{3}}$$

In the packing density limit, *N*Λ^3^/*V* → 1, μ^qv^ diverges, whereas μ^id^ → 0. It is common to express the chemical potential of a gas in terms of its pressure and a standard state pressure. Adopting the pressure, *p* ^qv^, in eq 5, we arrive at

16 $$\mu^{\mathrm{qv}}=p^{\mathrm{qv}}\Lambda^{3}+k_{B}T\ln\frac{N\Lambda^{3}}{V}$$

With the standard-state pressure, *p* ^◦^, (see e.g., pages 210–211 in ref 4),

17 $$p^{◦}=\frac{k_{B}T}{\Lambda^{3}}$$

μ^qv^ can be rewritten as

18 $$\mu^{\mathrm{qv}}=k_{B}T\left(\frac{p^{\mathrm{qv}}}{p^{◦}}+\ln\frac{N\Lambda^{3}}{V}\right)$$

### Bragg-Williams Equation of State

For the Bragg-Williams, BW, equation of state, the qv equation of state is extended with intermolecular interactions.^13^ Adopting the Bragg-Williams mean-field approximation for a lattice gas, the interaction contribution to the internal energy, $U_{\mathrm{inter}}^{\mathrm{BW}}$ , becomes

19 $$U_{\mathrm{inter}}^{\mathrm{BW}}\approx \frac{-u_{\mathrm{dis}}N^{2}}{M}$$

The energy, *u*_dis_, is the energy per particle required to remove a particle from a fully occupied lattice, and was introduced as^13^

20 $$u_{\mathrm{dis}}=\frac{-zw}{2}$$

where *z* is the number of nearest-neighbor lattice points and *w* is the pair interaction energy for nearest neighbors. Thereby are *z* and *w* merged into one parameter. Again, the volume of the system, *V*, is proportional to the number of lattice points, *M*, as

21 V=bM

where *b* is the volume of a single lattice point. In the quantum-volume Bragg-Williams (qv-BW) equation of state, *b* = Λ^3^, where Λ is given in eq 1. In the canonical ensemble, the volume is one of the independent variables, and therefore the number of lattice points needs to be dependent on temperature, *M*(β), so that *V* = *b*(β) *M*(β), i.e., the volume of the system is independent of the temperature. Although *M*(β) is a well-defined property because both *V* and *b*(β) are known, it will not appear explicitly in the equations to follow since it will be replaced *M*(β) = *V*/*b*(β).

Inserting eq 21 as M = *V*/*b* in eq 19, and for the Helmholtz free energy, *F* ^BW^, utilizing both $F^{\mathrm{BW}}=U_{\mathrm{inter}}^{\mathrm{BW}}-TS_{\mathrm{conf}}^{\mathrm{qv}}$ , where ln *Q* ^qv^ is given in eq 3, and *F* ^BW^ = −*k*_B_ *T* ln *Q* ^BW^, where *Q* ^BW^ is the partition function of the Bragg-Williams equation of state, we arrive at^13^

22 $$\ln Q^{\mathrm{BW}}=\ln\frac{V^{N}}{N!}+N\ln\frac{1}{b}+N\left(\left(1-\frac{V}{Nb}\right)\ln\left(1-\frac{Nb}{V}\right)-1\right)+\beta u_{\mathrm{dis}}\frac{N^{2}b}{V}$$

which, for *b* = Λ^3^, is the basis for the following derivations. We regard here a cubic lattice, which follows from that we regard the translational contribution to the molecular partition function as being derived from the three-dimensional particle-in-a-box model. Regarding the molecular partition function, the translational contribution is included in eq 22 for *b* = Λ^3^, because it has been merged with the configurational entropy, whereas the rotational, vibrational and electronic contributions are ignored in this work. Vibrational contribution should be included when polyatomic molecules are considered, which should be modeled by that a molecule occupies several lattice points since each lattice point is associated with an (atomic) mass, see eq 1. For the electronic contribution to the partition function, closed-shell systems are considered which can be assumed to be only in their nondegenerate ground state. An important aspect of eq 22 and its potential extensions is that an ideal gas is obtained as a limiting case, i.e., *b* = Λ^3^, *u*_dis_ = 0 and *N*/*V* → 0, as exercised elsewhere.^13^

#### Hard-Core Extension

For the qv-BW model in eq 22, the choice is *b* = Λ^3^ in line with the suggestion by Yu,^14^ which works well for an ideal gas and for a nonideal gas where interactions are ignored apart from the excluded volume Λ^3^ per particle as in the qv equation of state. In conjunction with an interaction energy between particles in adjacent lattice points, the qv-BW model, however, fails. The reason is that the distance Λ between the center of two adjacent lattice points is far too small compared to the energy minimum distance in the interaction potential, which is the distance for which we have the interaction energy *w*, as discussed also in our previous work.^13^ We therefore need to extend the qv-BW model so that the volume of a lattice point matches the distance corresponding to the potential energy minimum.

One approach would be to follow the work by Tonks that obtained the partition function for a one-dimensional system of hard spheres exactly, and in line with this solution suggested approximate solutions for two- and three-dimensional systems, however, in the high-density limit.^23^ If we instead turn the problem around, quantum corrections to hard-sphere models have been studied.^24–27^ For the model by Hemmer and Jancovici,^24,25^ the leading term in their expansion may be written as

23 $$\sigma_{\mathrm{eff}}\approx \sigma_{d}+c\Lambda$$

where σ_d_ is the hard-sphere diameter, *c* is a prefactor, and σ_eff_ is the quantum-corrected hard-sphere diameter. Higher-order corrections to eq 23 would include also a density dependence on σ_eff_.^27^ For the volume of a lattice point, we may adopt in line with the Hemmer-Jancovici model

24 $$b\left(\beta \right)={\left(\sigma_{d}+\Lambda (\beta )\right)}^{3}$$

and include it in eq 22. The prefactor is chosen as *c* = 1 to get the result for an ideal gas as a limiting case in eq 22, whereas $c=1/2\sqrt{2}$ for hard spheres. Regarding *b*′(β), however,

25 $$b^{\prime }\left(\beta \right)=\frac{\partial b}{\partial \beta }=\frac{3\Lambda }{2\beta }{\left(\sigma_{d}+\Lambda \right)}^{2}$$

it is realized that the correct high-temperature limits for the internal energy and the isochoric heat capacity are not obtained. Since Λ → 0 when *T* → ∞, both *U* and *C*_*V*_ will approach zero at high temperatures since they will include terms like *b*′(β). For energy contributions with infinitely many available energy levels, as for example the translational energy, *U* and *C*_*V*_ should in the limit *T* → ∞ approach their ideal gas values. It is only for systems with a finite number of energy levels, as for example the two-state Schottky model, that *C*_*V*_ approaches zero at high temperatures.

We can write the virial expansion of the pressure in terms of the molar volume, *V*_m_ = *V*/*N*, as

26 $$p=\frac{k_{B}T}{V_{m}}\left(1+\frac{B}{V_{m}}+...\right)$$

where *B* is the second virial coefficient. Again, utilizing the Maclaurin series, ln (1-*x*) ≈ - *x* - *x* ^2^/2, the pressure in eq 5 may be written as

27 $$\begin{array}{ll} p^{\mathrm{qv}} & \approx -\frac{k_{B}T}{\Lambda^{3}}\left(-\frac{\Lambda^{3}}{V_{m}}-\frac{{\left(\Lambda^{3}\right)}^{2}}{2V_{m}^{2}}\right)\\ & =\frac{k_{B}T}{V_{m}}\left(1+\frac{\Lambda^{3}}{2V_{m}}\right) \end{array}$$

identifying the virial coefficient as

28 $$B^{\mathrm{qv}}=\frac{\Lambda^{3}}{2}$$

It has been shown for an ideal bose gas that^28^

29 $$B\propto \Lambda^{3}$$

and the derivations for a nonideal bose gas,^29–31^ can essentially be expressed as

30 $$B\propto \Lambda^{3}f\left(T,V_{m}\right)$$

where *f*(*T*, *V*_m_) can be derived for some limiting cases. The subsequent appearance of models like the Hemmer-Jancovici expansion in eq 23 arises from the preassumption of a hard sphere, or any other model force field like the Lennard-Jones potential, as a suitable zeroth order term in a perturbation expansion. It is therefore justified to use a model for our *b* parameter that is proportional to Λ^3^, but *f*(*T*, *V*_m_) is unknown and will be largely determined by additional quantum effects at low temperatures. A simple model that gives the correct limiting behavior at high temperatures is, however,

31 $$b={\left(\sigma \Lambda \right)}^{3}$$

where σ is regarded as a scaling parameter equal to one in the ideal gas limit. We also note that since σ is just a constant prefactor,

32 $$b^{\prime }\left(\beta \right)=\frac{3{\left(\sigma \Lambda \right)}^{3}}{2\beta },\mathrm{and},b^{\prime \prime }\left(\beta \right)=\frac{3{\left(\sigma \Lambda \right)}^{3}}{4\beta^{2}}$$

and by comparison to the derivations leading to eqs 7 and 12, it is found that Λ^3^ can simply be replaced by (σΛ)^3^ in all the equations for the qv equation of state. Since eq 31 is only one of several possibilities, we denote this model the qv-BW-σΛ equation of state. The partition function for the qv-BW-σΛ equation of state is thus given by eq 22 with *b* = (σΛ)^3^. Still, in the remaining part, equations will first be derived for a general *b*(β) and subsequently we apply *b* = (σΛ)^3^.

#### Critical Point

The pressure, *p* ^BW^, is given from eq 22 with *F* ^BW^ = −*k*_B_ *T*ln*Q* ^BW^ as^13^

33 $$p^{\mathrm{BW}}=-{\left(\frac{\partial F^{\mathrm{BW}}}{\partial V}\right)}_{T,N}=-\frac{k_{B}T}{b}\ln\left(1-\frac{Nb}{V}\right)-\frac{u_{\mathrm{dis}}bN^{2}}{V^{2}}$$

which is rewritten in terms of the molar volume *V*_m_ = *V*/*N* (a proper molar volume, i.e., in units per mol, can be obtained by replacing *k*_B_ with the gas constant, R = *k*_B_ *N*_A_ where *N*_A_ is Avogadro’s constant) as

34 $$p^{\mathrm{BW}}=-\frac{k_{B}T}{b}\ln\left(1-\frac{b}{V_{m}}\right)-\frac{u_{\mathrm{dis}}b}{V_{m}^{2}}$$

The conditions for the critical point are given as (see e.g., page 508 in ref 4),

35 $${\frac{\partial p^{\mathrm{BW}}}{\partial V_{m}}|}_{c}=\frac{-k_{B}T_{c}}{\left(V_{m,c}-b_{c}\right)V_{m,c}}+\frac{2u_{\mathrm{dis}}b_{c}}{V_{m,c}^{3}}=0$$

and

36 $${\frac{\partial^{2}p^{\mathrm{BW}}}{\partial V_{m}}|}_{c}=\frac{k_{B}T_{c}\left(2V_{m,c}-b_{c}\right)}{{\left(V_{m,c}-b_{c}\right)}^{2}V_{m,c}^{2}}-\frac{6u_{\mathrm{dis}}b_{c}}{V_{m,c}^{4}}=0$$

where the notation indicates that the derivatives are evaluated at the critical point denoted by c. These two conditions together with the pressure in eq 34 leads to the following relatively simple relations for the critical point,

37 $$k_{B}T_{c}=\frac{1}{2}u_{\mathrm{dis}}$$

38 $$V_{m,c}=2b_{c}$$

and

39 $$p_{c}=\frac{\left(2\ln2-1\right)u_{\mathrm{dis}}}{4b_{c}}$$

where *b*_c_ denotes the lattice point volume evaluated at β_c_ = 1/*k*_B_ *T*_c_. The compressibility factor at the critical point, *Z*_c_, becomes

40 $$Z_{c}=\frac{p_{c}V_{m,c}}{k_{B}T_{c}}=2\ln2-1\approx 0.386$$

i.e. *Z*_c_ is independent of both *b*_c_ and *u*_dis_. In comparison, simple fluids have typical values for *Z*_c_ in the range 0.28–0.30, whereas the value for the van der Waals equation of state is *Z*_c_ = 3/8 = 0.375.^32^ The explicit results for the qv-BW-σΛ equation of state becomes

41 $$V_{m,c}=2{\left(\sigma \Lambda_{c}\right)}^{3}$$

and

42 $$p_{c}=\frac{\left(2\ln2-1\right)u_{\mathrm{dis}}}{4{\left(\sigma \Lambda_{c}\right)}^{3}}$$

in addition to the critical temperature in eq 37. Λ_c_ denotes that Λ in eq 1 is calculated at *T*_c_. Here, we also find the experimental connection for the parameters, σ can be obtained directly from *V*_m,c_ in eq 41 and *u*_dis_ from *T*_c_ in eq 37.

#### Internal Energy, Heat Capacity and Chemical Potential

When considering the internal energy, *U*, and the isochoric heat capacity, *C*_*V*_, we can for the configurational entropy terms use the results for the qv equation of state in eq 7 and 9. The internal energy for the BW model is obtained from the partition function in eq 22 as

43 $$U^{\mathrm{BW}}=-{\left(\frac{\partial \ln Q^{\mathrm{BW}}}{\partial \beta }\right)}_{V,N}=-\frac{Vb^{\prime }\ln\left(1-\frac{Nb}{V}\right)}{b^{2}}-\frac{N^{2}u_{\mathrm{dis}}}{V}\left(\beta b^{\prime }+b\right)$$

and the isochoric heat capacity becomes

44 $$\begin{array}{ll} C_{V}^{\mathrm{BW}} & =-k_{B}\beta^{2}{\left(\frac{\partial U^{\mathrm{BW}}}{\partial \beta }\right)}_{V,N}=k_{B}\beta^{2}(\frac{V\left(b^{\prime \prime }b-2b^{\prime 2}\right)}{b^{3}}\ln\left(1-\frac{Nb}{V}\right)\\ & -\frac{Nb^{\prime 2}}{b^{2}\left(1-\frac{Nb}{V}\right)}+\frac{N^{2}u_{\mathrm{dis}}}{V}\left(\beta b^{\prime \prime }+2b^{\prime }\right)) \end{array}$$

For the qv-BW-σΛ equation of state, we thus only need to derive terms including the interaction energies. We get

45 $$\beta b^{\prime }+b=\frac{5}{2}{\left(\sigma \Lambda \right)}^{3}$$

so that *U* in eq 43 for the qv-BW-σΛ model becomes

46 $$U=-\frac{3k_{B}TV\ln\left(1-\frac{N{\left(\sigma \Lambda \right)}^{3}}{V}\right)}{2{\left(\sigma \Lambda \right)}^{3}}-\frac{5N^{2}u_{\mathrm{dis}}{\left(\sigma \Lambda \right)}^{3}}{2V}$$

where the configurational part is obtained from eq 7 simply by replacing Λ^3^ by (σΛ)^3^. The molar internal energy, *U*_m_, with *V*_m_ = *V*/*N*, is thus

47 $$U_{m}=\frac{U}{N}=-\frac{3k_{B}TV_{m}\ln\left(1-\frac{{\left(\sigma \Lambda \right)}^{3}}{V_{m}}\right)}{2{\left(\sigma \Lambda \right)}^{3}}-\frac{5u_{\mathrm{dis}}{\left(\sigma \Lambda \right)}^{3}}{2V_{m}}$$

The ideal gas result, *U*_m_ = 3*k*_B_ *T*/2, is obtained by setting σ = 1, *u*_dis_ = 0, and using the Maclaurin series ln­(1-*x*) ≈ -*x*. For the heat capacity, we need

48 $$\beta b^{\prime \prime }+2b^{\prime }=\frac{15{\left(\sigma \Lambda \right)}^{3}}{4\beta }$$

which is combined with the qv expression for *C*_*V*_ in eq 12 to arrive at the result for the qv-BW-σΛ model,

49 $$C_{V}=-\frac{15k_{B}V}{4{\left(\sigma \Lambda \right)}^{3}}\ln\left(1-\frac{N{\left(\sigma \Lambda \right)}^{3}}{V}\right)-\frac{9Nk_{B}}{4\left(1-\frac{N{\left(\sigma \Lambda \right)}^{3}}{V}\right)}+\frac{15k_{B}\beta N^{2}u_{\mathrm{dis}}{\left(\sigma \Lambda \right)}^{3}}{4V}$$

and the molar *C*_*V*_ becomes

50 $$C_{V,m}=-\frac{15k_{B}V_{m}}{4{\left(\sigma \Lambda \right)}^{3}}\ln\left(1-\frac{{\left(\sigma \Lambda \right)}^{3}}{V_{m}}\right)-\frac{9k_{B}}{4\left(1-\frac{{\left(\sigma \Lambda \right)}^{3}}{V_{m}}\right)}+\frac{15k_{B}\beta u_{\mathrm{dis}}{\left(\sigma \Lambda \right)}^{3}}{4V_{m}}$$

Finally, the chemical potential, μ, for the qv-BW-σΛ equation of state is obtained from eq 14 and ([Disp-formula eq22]) as

$$\mu =-k_{B}T{\left(\frac{\partial \ln Q^{\mathrm{BW}}}{\partial N}\right)}_{T,V}=-k_{B}T\ln\left(\frac{V}{N{\left(\sigma \Lambda \right)}^{3}}-1\right)-\frac{2Nu_{\mathrm{dis}}{\left(\sigma \Lambda \right)}^{3}}{V}$$ 51

## Results and Discussion

### Critical Point

The parameters for the qv-BW-σΛ equation of state are here determined from experimental data of the critical point collected in Table 1 (see appendix 5 in ref 33) for a set of model systems for which we assume that they occupy only one lattice point adopting a hydrogen-suppressed assumption. For molecules, we will within this model in general assume that they occupy several lattice points, one for each non-hydrogen atom.

**Table 1:** Selected Experimental Data of the Critical Point (See Appendix 5 in Ref 33)

	*T*_c_/K	*V*_m,c_/cm^3^ mol^–1^	*p*_c_/bar
Ne	44.40	41.7	27.6
Ar	150.8	74.9	48.7
Kr	209.4	91.2	55.0
Xe	289.7	118	58.4
CH_4_	190.5	99	46.1

The parameters *u*_dis_ and σ are determined to exactly reproduce the experimental *T*_c_ and *p*_c_ for the qv-BW-σΛ model using eqs 37 and 39, respectively, as presented in Table 2. The argument for parametrizing *T*_c_ and *p*_c_, and not *V*_m,c_, is that the critical molar volume is more difficult to determine in an accurate way experimentally because of the large fluctuations in the volume close to the critical point (see e.g., page 183 in ref 34). In Table 2, we find that *V*_m,c_ is consistently a factor of around 4/3 larger than the experimental value. The reason is that the compressibility factor in eq 40 is, as the compressibility factor for the van der Waals equation of state, around a factor 4/3 larger than the experimental compressibility factors that are very similar for these systems. One can imagine other models for *b*(β) with an improved compressibility factor. An argument for using experimental data at the critical point is that it is a region where nonideal contributions actually are significant. At ambient conditions, the included systems are very close to ideal gases, and the nonideal contributions modeled by our parameters can safely be ignored.

**Table 2:** Results for the Qv-BW-σΛ Model

	*u*_dis_/kJ mol^–1^	Λ_c_/Å	σ	σΛ_c_/Å	*V*_m,c_/cm^3^ mol^–1^	$V_{m,c}/V_{m,c}^{\mathrm{exptl}}$
Ne	0.74	0.58	6.00	3.50	51.7	1.34
Ar	2.51	0.22	19.36	4.35	99.5	1.33
Kr	3.48	0.13	35.40	4.66	122.3	1.34
Xe	4.82	0.09	56.92	5.09	159.3	1.35
CH_4_	3.17	0.32	15.18	4.79	132.7	1.34

Quantum chemical results from the literature on the coupled-cluster level for the dimers corresponding to the systems included in Tables 1 and 2 are presented in Table 3.^35–39^ A comparison to quantum chemical data for dimer calculations is, however, not straightforward. The bond distance, *R*_e_, may be compared to σΛ_c_ keeping in mind that Λ is dependent on temperature. Also, the translational partition function are obtained at a high-temperature limit, and Λ diverges at *T* = 0 K. Anyway, comparing σΛ_c_ in Table 2 with *R*_e_ in Table 3, σΛ_c_ is consistently around 0.5 Å larger than *R*_e_ for the rare gas atoms whereas it is more than 1 Å larger for methane. This comparison is also consistent with that that the model value for *V*_m,c_ in Table 2 is larger than the corresponding experimental value. Neither *u*_dis_ can be compared directly to the dimer interaction energy, *V*_int_, since it models an averaged internal energy for *z* neighbors, and its absolute value should thus be considerably larger than the dimer interaction energy. Also, *V*_int_ is obtained at 0 K and for example, the zero-point vibrational energy is ignored. Still, comparing *u*_dis_ in Table 2 and *V*_int_ in Table 3, the rare gas systems follow the same relative trend with around a factor two difference, but again with a deviation for methane. To summarize, although the parameter values can be improved by regarding more exponential data, they seem reasonable and sufficient for a model study. Also, the accuracy of parameter values is in general strongly dependent on the accuracy of the underlying model.

**Table 3:** Quantum Chemical Results of Dimers on the Coupled-Cluster Level

	*R*_e_/Å	*V*_int_/kJ mol^–1^	Reference
Ne_2_	3.09	0.35	^35^
Ar_2_	3.78	1.17	^36^
Kr_2_	4.06	1.67	^37^
Xe_2_	4.36	2.35	^38^
(CH_4_)_2_	3.6	2.26	^39^

### Excluded-Volume Term

Since the hard-core potential, σΛ, is dependent on temperature, the temperature dependence for both Λ and σΛ are shown in Figures 1 and 2, respectively, for the model systems in Table 2. Since the temperature dependence of Λ is $1/\sqrt{T}$ , it approaches zero very slowly, and over a short temperature interval, it can in principle be regarded as independent of temperature. For Λ in Figure 1, the order of the systems depends entirely on their mass with the lightest system, methane, on the top, and xenon at the bottom. For σΛ in Figure 2, the rare gas atoms appear in the opposite order with xenon at the top and neon at the bottom in line with *R*_e_ in Table 3. Methane is very similar to krypton.

**Figure 1:**
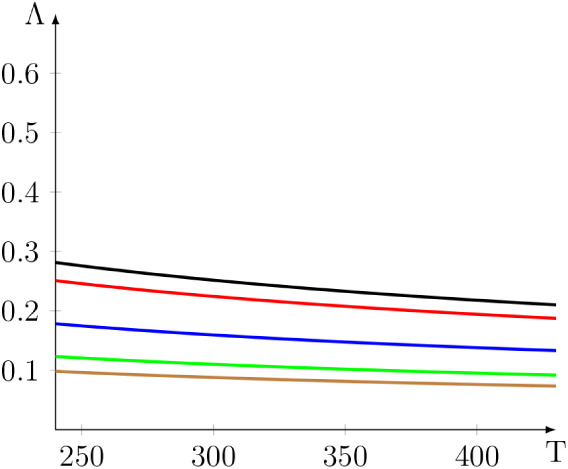
Thermal wavelength, Λ (Å), as a function of the temperature, *T* (K). Color code: Ne: red, Ar: blue, Kr: green, Xe: brown, CH_4_: black.

**Figure 2:**
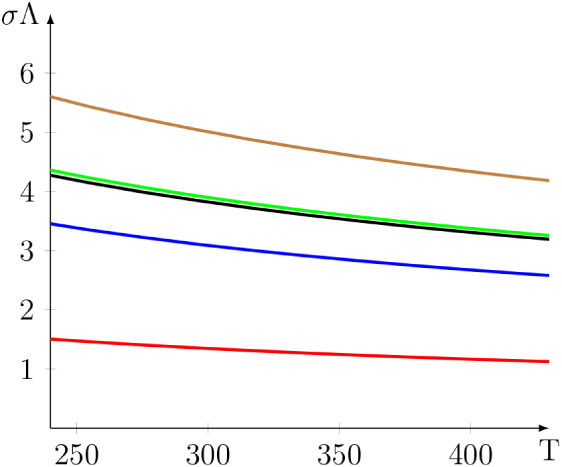
Hard-core potential, σΛ (Å) as a function of the temperature, *T* (K). Color code: Ne: red, Ar: blue, Kr: green, Xe: brown, CH_4_: black.

It is noted (not shown in figures but one may compare Λ^3^ to the results presented below for (σΛ)^3^ for Ne, that behaves like an ideal gas) that Λ is so small for all systems that Λ^3^/*V*_m,c_ can be ignored in eq 7 apart from at very low temperatures, less than 20 K, where the model anyway would not be valid because of quantum effects. For example, Λ in eq 1 is obtained from the translational partition function, where a sum over states is approximated by an integral (for corrections see e.g., ref 40). Therefore, the qv equation of state should mainly be regarded as a theoretical framework, and actual results are therefore presented only for the qv-BW-σΛ equation of state.

### Internal Energy

For illustrating the temperature dependence of the molar internal energy in eq 47, we adopt the experimental molar volumes at the critical point, *V*_m,c_, in Table 1 which is sufficient to demonstrate the main features of the model. At ambient conditions, only the ideal contribution would be visible. In Figure 3, the configurational (kinetic) contribution to the molar internal energy is presented for the qv-BW-σΛ equation of state, which converge toward 3*k*_B_ *T*/2 at high temperatures. All curves are above the ideal gas line with neon essentially overlapping with the ideal gas. For the temperature scale shown, only xenon is approaching the divergence at the packing density limit, but all systems will do that at lower temperatures. Apart from neon, the nonideal contribution arising from the configurational entropy is significant for all systems.

**Figure 3:**
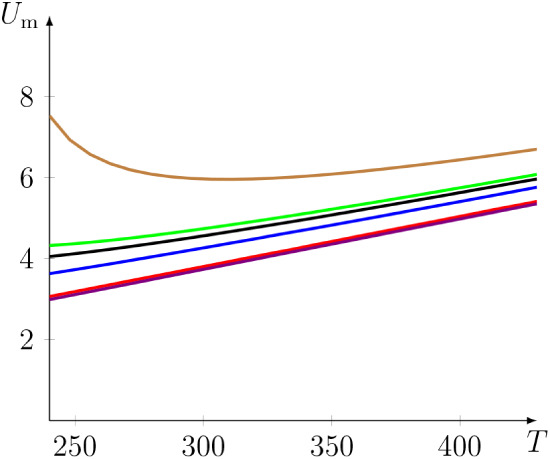
Configurational contribution to the molar internal energy, *U*_m_ (kJ/mol), as a function of the temperature, *T* (K), for the qv-BW-σΛ model. Color code: Ne: red, Ar: blue, Kr: green, Xe: brown, CH_4_: black, ideal gas: violet.

In Figure 4, the interaction term to *U*_m_ is presented. For all systems, this term will eventually reach zero at high temperatures, again with neon showing an ideal behavior over the entire presented temperature scale. The curves appear in the expected order with xenon at the bottom, and again with methane fairly close to krypton. In Figure 5, the total internal energy is presented. As expected from Figures 3 and 4, neon is also here very close to an ideal behavior. As in Figure 3, all systems will approach ideal behavior at high temperatures reaching the value 3*k*_B_ *T*/2. Within the presented temperature scale, only xenon shows a minimum, but all systems will have a minimum and diverge to plus infinity at the packing density limit. In Figure 6, the internal energy is given as a function of the molar volume at 300 K. Its purpose is to show that all systems reach ideal behavior long before *V*_m_ ≈25·10^3^ cm^3^/mol, as obtained from the ideal gas law at ambient conditions.

**Figure 4:**
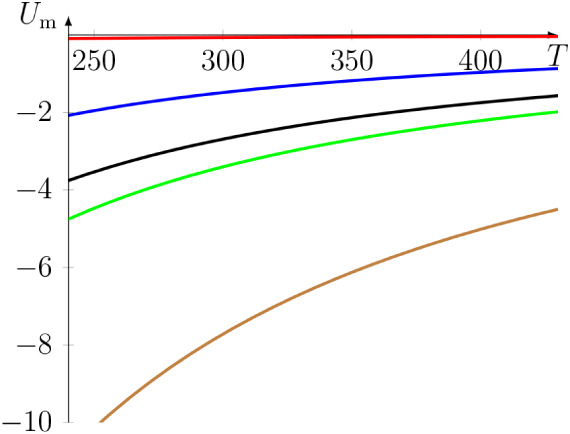
Interaction energy contribution to the molar internal energy, *U*_m_ (kJ/mol), as a function of the temperature, *T* (K) for the qv-BW-σΛ model. Color code: Ne: red, Ar: blue, Kr: green, Xe: brown, CH_4_: black.

**Figure 5:**
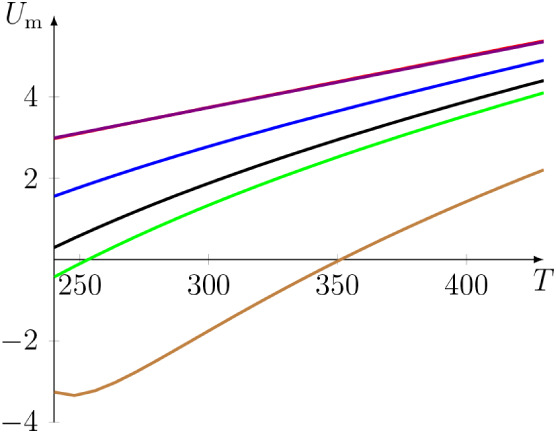
Molar internal energy, *U*_m_ (kJ/mol), as a function of the temperature, *T* (K), for the qv-BW-σΛ model. Color code: Ne: red, Ar: blue, Kr: green, Xe: brown, CH_4_: black, ideal gas: violet.

**Figure 6:**
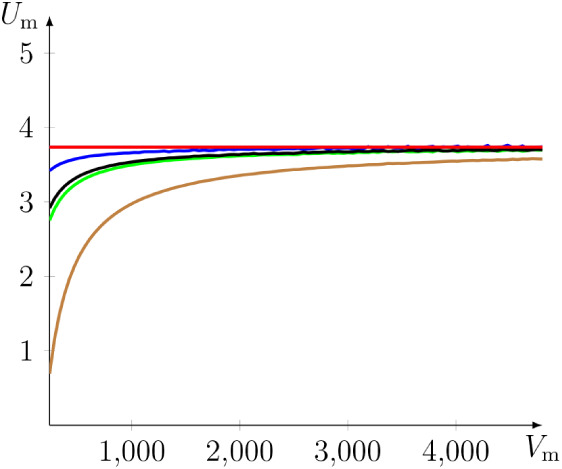
Molar internal energy, *U*_m_ (kJ/mol), at 300 K as a function of the molar volume, *V*_m_ (cm^3^/mol), for the qv-BW-σΛ model. Color code: Ne: red, Ar: blue, Kr: green, Xe: brown, CH_4_: black, ideal gas: violet.

### Isochoric Heat Capacity

It has been demonstrated experimentally that *C*_*V*_ has a divergence at the critical point with a critical exponent, α = 0.11.^41–43^ *C*_*p*_ is related to *C*_*V*_ as (see e.g., page 105 in ref 44),

52 $$C_{p}-C_{V}=\frac{\alpha_{p}^{2}VT}{\kappa_{T}}=\frac{T{\left(\frac{\partial p}{\partial T}\right)}_{V}^{2}}{{\left(\frac{\partial p}{\partial V}\right)}_{T}}$$

where α_*p*_ is the thermal expansion coefficient and κ_*T*_ is the isothermal compressibility. The condition for the critical point in eq 35 appears in the denominator of *C*_*p*_ - *C*_*V*_, and therefore it diverges at the critical point. We also note that in general *C*_*V*_ > 0, κ_*T*_ > 0 and therefore *C*_*p*_ > *C*_*V*_ (see e.g., pages 35–36 in ref 45). The isothermal compressibility, κ_*T*_, is defined as (see e.g., page 176 in ref 46),

53 $$\kappa_{T}=-\frac{1}{V}{\left(\frac{\partial V}{\partial p}\right)}_{T}=-\frac{1}{V}\frac{1}{{\left(\frac{\partial p}{\partial V}\right)}_{T}}$$

and κ_*T*_ and therefore also *C*_*p*_ diverge at the critical point with a critical exponent, γ = 1. We also note that κ_*T*_ can be written in terms of volume fluctuations as (see e.g., pages 37–38 in ref 47),

54 $$\kappa_{T}=\beta \frac{\left\langle V^{2}\right\rangle -\langle V\rangle^{2}}{\left\langle V\right\rangle }$$

where the brackets ⟨···⟩ denote ensemble averages, in this case the *NpT*-ensemble. Therefore, it is the divergence in volume fluctuations that gives a divergence for both *C*_*p*_ and κ_*T*_. *C*_*V*_, on the other hand, depends only on energy fluctuations (see e.g., pages 65–66 in ref 45), and it only has a weak divergence at the critical point (see also discussions on pages 105–106 in ref 44. and on page 181 in ref 34).^43,48^ Experimentally, it is the specific heat, i.e., ρ_N_ *C*_*V*_ where ρ_N_ is the number density, that normally is measured,^49^ e.g., for neon,^50^ argon,^51^ and xenon^52^ and it has been argued that it is the density and not the heat capacity that is the origin of the divergence of the specific heat at the critical point.^53,54^ The heat capacity at supercritical conditions and near the critical point have also been studied by molecular dynamics simulations.^55,56^

Although the purpose of the development of most equations of state used for liquids has not been on accurate heat capacities, heat capacities of a variety of equations of state have been investigated and it was concluded that improvements are needed in regions where the ideal gas term is not dominating.^57,58^ Commonly, a crossover term has been included to account for the divergence at the critical point according to renormalization group theory.^59^

We will therefore in more detail investigate the temperature dependence around the critical point for the heat capacity of the qv-BW-σΛ model in eq 50. In Figure 7, the configurational contribution to *C*_*V*_ is presented. As for the internal energy, neon behaves as an ideal gas with a constant molar *C*_*V*_ of 3*k*_B_/2. At the other end, xenon has the strongest temperature dependence since it is the system closest to the packing density limit. The interaction contribution to *C*_*V*_ is shown in Figure 8, with trends consistent with the findings for the internal energy *U*.

**Figure 7:**
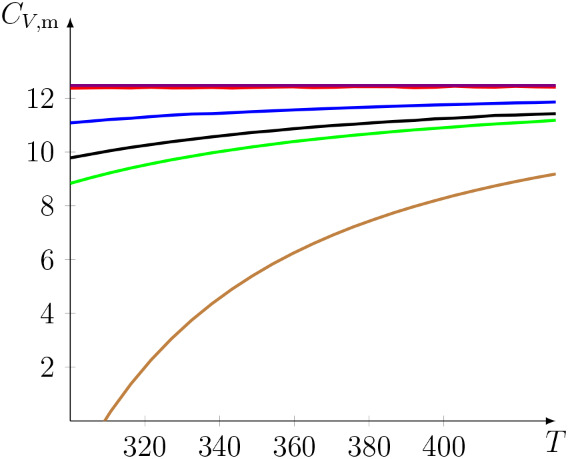
Configurational contribution to the molar isochoric heat capacity, *C*_*V*m_ (J/mol K), as a function of the temperature, *T* (K), for the qv-BW-σΛ model. Color code: Ne: red, Ar: blue, Kr: green, Xe: brown, CH_4_: black, ideal gas: violet.

**Figure 8:**
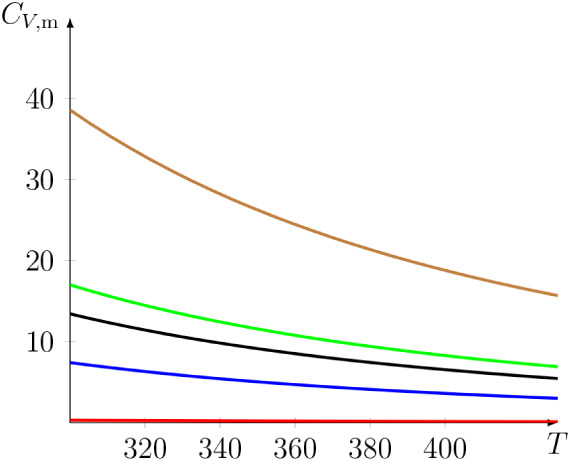
Interaction energy contribution to the molar isochoric heat capacity, *C*_*V*m_ (J/mol K), as a function of the temperature, *T* (K), for the qv-BW-σΛ model. Color code: Ne: red, Ar: blue, Kr: green, Xe: brown, CH_4_: black.

The total *C*_*V*_ is shown over a wider temperature range in Figure 9. Within this temperature range, all systems apart from neon show a maximum and then a rapid decrease toward a divergence at the packing density limit. The critical temperature, *T*_c_ is included for each system, and it is found for all systems that the maximum of *C*_*V*_ is close to *T*_c_, but not exactly at *T*_c_. Neon will also have a similar maximum, but close to its *T*_c_ at 44.40 K. The value of the heat capacity at the critical point is independent of the parameter values and thereby of the system, and is obtained as

55 $$C_{V,m,c}=\left(\frac{15}{2}\ln\left(2\right)-\frac{3}{4}\right)k_{B}\approx 37\;J/\mathrm{mol K}$$

explaining why the maxima appear at approximately the same value of the heat capacity for all systems. Experimentally, a divergence is expected at *T*_c_, but that cannot be achieved with a mean-field approach as the one developed here. It is still intriguing that we find a maximum of the isochoric heat capacity close to *T*_c_, and it is the most promising result of the model so far. Apart from its vicinity to the critical temperature, it is, however, not evident from the model that it reflects a critical behavior, and it can equally well be an effect of a proper inclusion of the packing-density limit.

**Figure 9:**
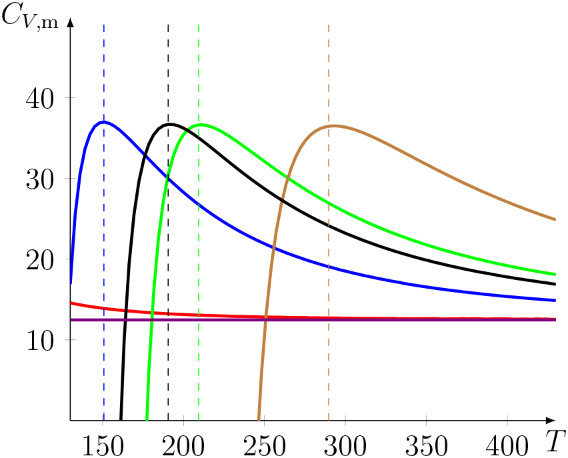
Molar isochoric heat capacity *C*_*V*m_ (J/mol K) as a function of the temperature *T* (K) for the qv-BW-σΛ model. Color code: Ne: red, Ar: blue, Kr: green, Xe: brown, CH_4_: black, ideal gas: violet. The dashed lines indicate *T*_c_ for respective system.

Finally, in Figure 10, the heat capacity is shown as a function of the molar volume. As for the internal energy, ideality is reached long before ambient conditions.

**Figure 10:**
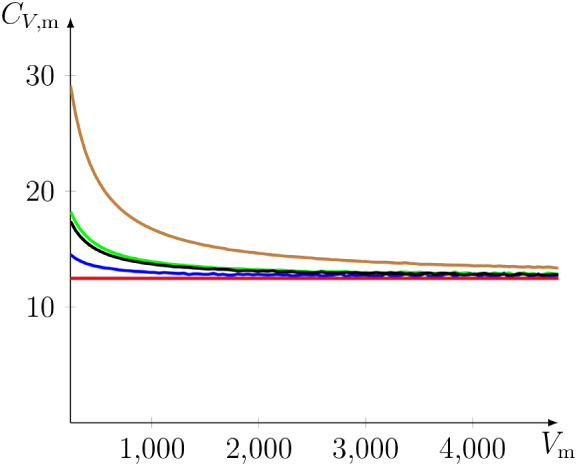
Molar isochoric heat capacity *C*_*V*m_ (J/mol K) as a function of the molar volume *V*_m_ (cm^3^/mol) for the qv-BW-σΛ model. Color code: Ne: red, Ar: blue, Kr: green, Xe: brown, CH_4_: black, ideal gas: violet.

## Summary and Conclusions

Based on our previous finding that the configurational and translational contributions to the partition function need to be merged for lattice models in molecular thermodynamics,^13^ quantum volume equations of state have been investigated. If a temperature-dependent excluded volume of Λ^3^ is included, but no additional interactions, a model has been established with a temperature of the internal energy and the isochoric heat capacity that gives the correct behavior at the packing-density as well as the ideal-gas limit.

The quantum-volume model was subsequently extended with an intermolecular interaction term and a temperature-dependent hard-core with a more realistic volume for a lattice point corresponding to the minimum of the intermolecular potential energy surface. For this equation of state, termed the qv-BW-σΛ equation of state, the critical point, internal energy, isochoric heat capacity, and chemical potential have been derived. The correct behavior in the ideal-gas, high-temperature, and packing-density limits were demonstrated. It was also demonstrated that the included systems actually behave as ideal gases at ambient conditions. Of particular interest is the finding that the isochoric heat capacity has a maximum close to, but not exactly at, the critical temperature. It is intriguing since experimentally it is found that the isochoric heat capacity has a weak divergence at the critical point.

From a more general perspective, the work presented here serves mainly as a basis for further developments developing analytical equations within molecular thermodynamics essentially on the textbook level. Further studies may include polyatomic molecules extending over several lattice points, mixtures, condensed phases, other ensembles than the canonical ensemble, and additional properties and processes including transport properties and chemical kinetics. Of particular interest would be to use the Flory–Huggins approach for polymers on polyatomic molecules.^3^ Also, it is expected that the maximum caliber framework can be directly applied on this model for obtaining analytical equations for nonequilibrium properties.^60,61^ Also, the accuracy at the low-temperature limit of the model should be exploited regarding the derivation of the translational partition function and other quantum effects.
